# Evaluation of Esthetic Results after Mass Removal with Elliptical Skin Excision Using Ultrasonography to Measure Skin Thickness

**DOI:** 10.3390/jcm13051467

**Published:** 2024-03-03

**Authors:** Sang Seok Woo, Hongki Gwak, Ki Hyun Kim, Jun Won Lee, Jai Koo Choi, Insuck Suh, Seong Hwan Kim

**Affiliations:** 1 Department of Plastic and Reconstructive Surgery, Kangnam Sacred Heart Hospital, Hallym University College of Medicine, Seoul 07441, Republic of Korea; ssw1218@gmail.com (S.S.W.); rlgus0608@hanmail.net (K.H.K.); ljw2014@hallym.or.kr (J.W.L.); jkchoi57@gmail.com (J.K.C.); sismdps@hallym.or.kr (I.S.); 2 Division of Breast and Thyroid Surgical Oncology, Department of Surgery, Hwahong Hospital, Suwon 16630, Republic of Korea; hkgwak@gmail.com

**Keywords:** benign cutaneous masses, cellular origin, tumor expansion, elliptical skin excision, mass removal, postoperative esthetics, ultrasonography

## Abstract

**Background:** The growth of benign cutaneous masses causes the overlaying skin to expand and become thinner, especially at the central, most projected point. In this retrospective study, a surgical technique comprising an elliptical skin excision was employed to account for these skin changes. **Methods:** This retrospective study enrolled 980 patients with benign masses. Preoperatively, all patients underwent ultrasonography to evaluate the mass depth and thickness of the attached skin, and mass excision was performed using the elliptical skin-excision method. The operative time was recorded, and complications and esthetic outcomes were assessed using the Cutometer^®^ and the modified Vancouver Scar Scale (mVSS) during 1- and 3-month follow-up visits. **Results:** The mean operative time (17.48 ± 3.46 min) was significantly shorter than that of conventional methods (*p* < 0.05). Cutometer parameters showed no significant differences from those of intact skin. The average mVSS scores were 5.21 ± 1.42 and 3.50 ± 1.79 at 1- and 3-month follow-ups, respectively. **Conclusions:** Mass excision with an elliptical skin attachment resulted in improved esthetic results and easy removal. The attached skin enabled convenient handling without damaging the capsule or other adjacent structures, leaving a thick dermis on both wound edges. Thus, this technique resulted in minimal scarring.

## 1. Introduction

Skin pathologies, including benign cutaneous and soft tissue masses such as epidermal cysts, lipomas, dermoid cysts, and pilomatricomas, are diverse conditions that often require surgical intervention [1]. While benign in nature, these masses can have considerable esthetic and psychological effects on patients, especially if their location is conspicuous. Recently, surgery has aimed to achieve not only complete mass removal but also minimal scar formation. The primary goal of surgical management is the complete removal of these masses with rapid healing without other complications, such as inflammation, which also leads to less scarring [2,3]. Historically, numerous techniques have been employed for this purpose, including direct excision, suction, topical corticosteroid administration, and various types of minimally invasive surgery [3,4,5,6,7,8]. Each of these methods has strengths and limitations, including recurrence due to incomplete removal, delayed healing, wound dehiscence, and other complications that can eventually lead to unfavorable scar formation [3,9,10]. 

Among the different surgical techniques, elliptical skin excision has shown promise in its ability to manage benign cutaneous masses, particularly larger masses. The skin over the subcutaneous round or dome-shaped masses usually becomes thinner, especially at the central, most projected point. With ultrasonography, the character, size, and depth of masses can be examined, and the thickness of the attached skin can also be measured. The elliptical skin-excision technique involves the excision of the lesion with an elliptically shaped portion of the overlying skin and offers the advantage of ensuring complete lesion removal while minimizing the visibility of the resultant scar without dog-ear formation [3,10,11,12,13,14,15,16]. 

The present study aimed to evaluate the effectiveness, esthetic outcomes, and safety of the elliptical skin-excision technique for managing benign skin masses. We present our experiences and outcomes using this technique in a series of cases and discuss its potential advantages and drawbacks in comparison with those of alternative methods [2,3,4,5,6,7,8,9,10,11,12,13,14,15,16,17]. Through this analysis, we hope to provide valuable insights into the role of elliptical skin excisions in the management of benign skin pathologies.

## 2. Materials and Methods

This retrospective study had the approval of the Institutional Review Board of Kang-nam Sacred Heart Hospital (Institutional Review Board (IRB) number 2023-11-027). All procedures involving human participants were conducted in accordance with the ethical standards established by the Institutional and/or National Research Committee and the 1964 Declaration of Helsinki and its later amendments or comparable ethical standards.

### 2.1. Patient Selection and Surgical Methods

Clinical records were reviewed for 980 patients who underwent elliptical excision with mass attachment to remove skin lesions between January 2018 and April 2023. All patients (524 men and 456 women) were of Northeast Asian origin and were aged 19–84 years. Before surgery, all patients underwent ultrasonography to evaluate the depth of the mass and the thickness of the attached skin. Mass excision was performed using the same elliptical skin-excision method.

Patients were positioned appropriately according to the location of the masses, and the area of the mass was prepared and draped in a sterile fashion. An elliptical skin-excision line was drawn parallel to the relaxed skin tension line on the most expanded skin area, without crossing the protuberance area. A notable feature of this technique is that it includes the most protruded and expanded region of the skin attached to the mass within the elliptical excision. This served the dual purpose of removing the thin, potentially necrotic, skin overlying the mass and providing a handle for the mass that could be easily grabbed and manipulated during the operation. After marking the surgical site, 2% lidocaine mixed with 1:100,000 epinephrine was injected for hydrodissection. This not only helped in elevating the mass but also induced vasoconstriction, reducing intraoperative bleeding.

**Figure 1:**
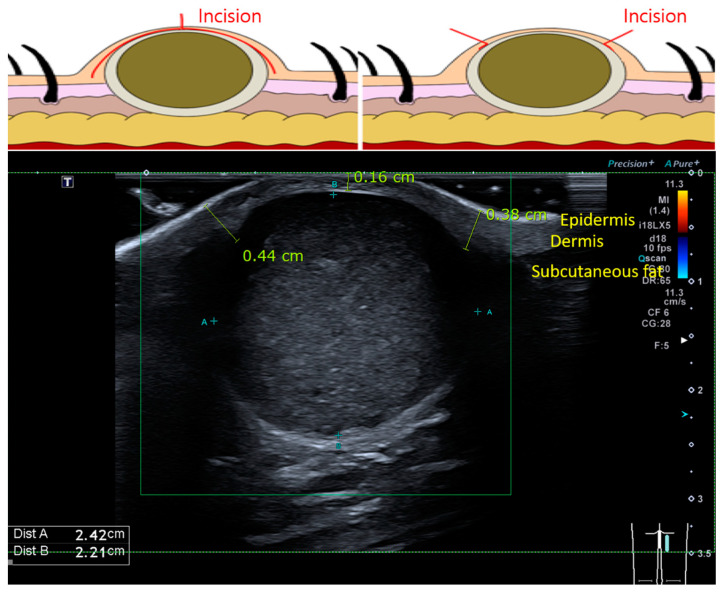
Schematic representation of a cutaneous mass and ultrasonographic result of an epidermal cyst. As the mass enlarges, the skin becomes thinner. The expanded skin is removed and attached to the masses. The operator does not need to touch the capsule of the mass, which is more prone to tearing.

**Figure 2:**
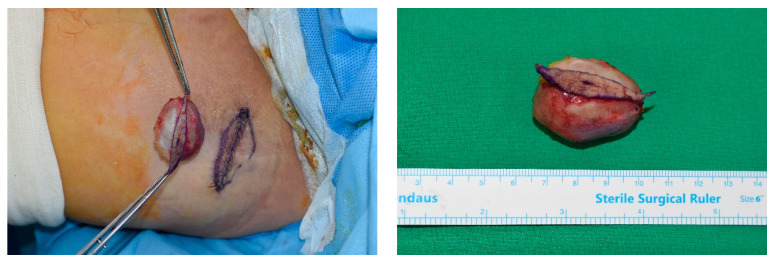
Clinical image of an elliptical incision performed for a patient with an epidermal cyst with a diameter of >3 cm. The operator can access the attached skin without damaging the capsule of the mass.

As the dermis over the mass became thinner than that of the adjacent normal area, a delicate incision was made to prevent injury to the underlying mass. The mass, along with the overlying skin, was then carefully dissected from the surrounding tissue using Metzenbaum scissors and electrocauterization, leaving any surrounding capsule intact (Figure 1 and Figure 2).

After removing the mass, wound closure was performed in the layers to prevent dead space and potential complications. The subcutaneous tissue was closed using buried absorbable sutures with Vicryl numbers 4-0, 5-0, and 6-0. The skin was then closed using simple interrupted or continuous skin sutures with Ethilon number 5-0 or 6-0, depending on the thickness and tension of the skin at the excision site. All patients were followed up with dressing changes every 2–3 days until the removal of the stitches, which typically occurred between 5 and 10 days postoperatively. 

### 2.2. Outcome Assessment

The study evaluated the clinical responses to treatment and complications. The operative time was recorded from the first incision until the last skin suture. Follow-up visits were scheduled at 1 and 3 months postoperatively to check for any potential complications and to assess the esthetic outcome of the surgery using both the Cutometer^®^ and the modified Vancouver Scar Scale (mVSS) in the first month, and the mVSS alone in the third month. 

### 2.3. Cutometer

The patients were evaluated in a comfortable environment with a room temperature of 23 ± 2 °C and a relative humidity of 35 ± 5%. Skin elasticity was measured using the Cutometer (MPA 580; Courage + Khazaka electronic GmbH, Cologne, Germany) [18]. The Cutometer measures the vertical deformation of the skin as it is pulled into the circular opening by a controlled vacuum. The time/strain mode was used with five consecutive cycles of 5 s of suction application followed by a 3 s period of relaxation. A measuring probe with a 2 mm diameter was utilized, and constant suction was applied at 500 mbar [18,19,20,21]. The study measured the average deformation of three points on the scar line (middle and both edges) and normal tissue in the same anatomic area adjacent to the related scar area [18,22,23].

**Figure 3:**
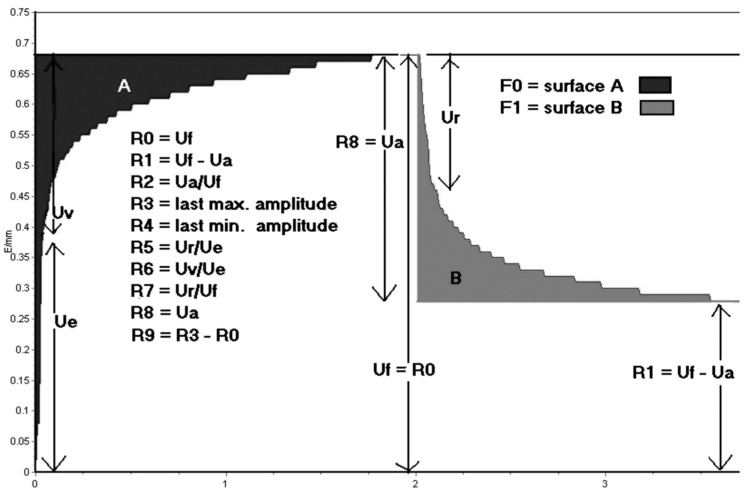
The skin deformation curve was obtained using the Cutometer. Uf represents the final distension at the end of the first vacuum period, while Ua represents the difference between the maximum deformation of the first vacuum period and the deformation after 1 s of normal pressure. Ur refers to the immediate relaxation within the first 0.1 s after the end of the initial vacuum period, the skin immediately distends (Ue) within the first 0.1 s of the vacuum period. Uv represents the difference between the deformation after 0.1 s and the maximal deformation of the first vacuum period. R denotes the residual deformation after the release of the first suction.

The study analyzed the difference between the maximum deformation of the skin during the first vacuum period and the deformation after 1 s of normal pressure (Ua), the immediate distension of the skin within the first 0.1 s of the first vacuum period (Ue), and the difference between the deformation after 0.1 s and the deformation after 1 s of normal pressure for each measured region. Skin deformation curves (Figure 3) provided values for Uv, Uf, and Ur. The embedded software was used to analyze the curves and extract parameters R0 to R9 from the measured values (Table 1) [18,19,21,23].

The following parameters were measured: final distension at the end of the first vacuum period (Uf), the difference between the maximum deformation of the first vacuum period and the deformation after 1 s of normal pressure (Ua), immediate relaxation within the first 0.1 s after the end of the first vacuum period (Ur), immediate distension of the skin within the first 0.1 s of the first vacuum period (Ue), and the difference between the deformation after 0.1 s and the maximal deformation of the first vacuum period (Uv).

### 2.4. mVSS

The scars were evaluated based on their vascularity, pigmentation, pliability, height, pain, and pruritus. Vascularity was rated on a scale of normal (0), pink (1), red (2), or purple (3), while pigmentation was rated on a scale of normal (0), hypopigmented (1), mixed pigmented (2), or hyperpigmented (3). Pliability was rated on a scale of normal (0), supple (1), yielding (2), firm (3), rope (4), or contracture (5), and height was rated on a scale of flat (0), <2 mm (1), 2–5 mm (2), or >5 mm (3). Pain and itching were scored on a scale from 0 to 2, with 0 indicating no symptoms and 2 indicating the need for medication. Each variable had between two and five possible scores. The total score ranged from 0 to 14, with a score of 0 reflecting normal skin [22].

### 2.5. Statistical Analysis

Independent t-tests were conducted to confirm the results by comparing the scar and normal tissues with the Cutometer parameters. The data analysis was performed using IBM SPSS Statistics for Windows (version 22.0; IBM Corp., Armonk, NY, USA). A *p*-value of less than 0.05 was considered statistically significant.

## 3. Results

### 3.1. Patients

The study enrolled 980 patients, comprising 524 men and 456 women. Men had a mean age of 58.1 ± 12.9 years, while women had a mean age of 43.2 ± 13.1 years. Most masses were located in the head and neck (*n* = 695, 70.9%) and trunk (*n* = 173, 17.7%) regions, with fewer masses in the extremities (*n* = 112, 11.4%). The most common pathology was epidermal cysts (*n* = 471, 48.1%), followed by lipomas (*n* = 131, 13.4%) and other benign masses such as pilomatricomas and dermoid cysts (*n* = 378, 38.5%). Basic patient demographics, including age, sex, and mass characteristics, are listed in Table 2.

### 3.2. Quantitative Evaluation (Objective)

For elliptical skin excision, the mass fixation technique was successful in all patients. The average operative time was 17.48 ± 3.46 min (range, 13–33 min) and the average mass size was 14.21 ± 7.5 mm (range, 3–43 mm) (Table 3). When comparing the surgical site to healthy skin, three parameters showed minimal differences, demonstrating a minimal loss of elasticity (*p* < 0.05). Those three parameters were R0, R3, and R8, representing the maximal skin distension (R0) as a measure of pliability; the maximum amplitude of the last suction curve after repeated suction (R3), indicating the increased tiring of the skin; and a marker of skin recovery (R8). The parameters showed 1.33 ± 0.30, 1.49 ± 0.30, and 0.92 ± 0.26, respectively, on the suture line compared to 1.52 ± 0.30, 1.65 ± 0.31, and 1.13 ± 0.25, respectively, on the normal skin. The parameters R1, R2, R4, R5, R6, R7, and R9 exhibited no significant difference (Table 4). 

**Figure 4:**
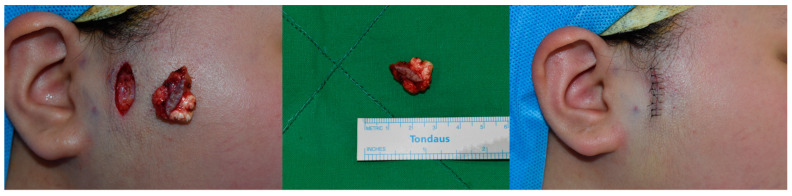
Elliptical skin excision performed on the preauricular pilomatricoma. The mass was successfully excised without damaging the adjacent structures and mass. The defect after mass excision was covered without dog-ear deformity.

**Figure 5:**
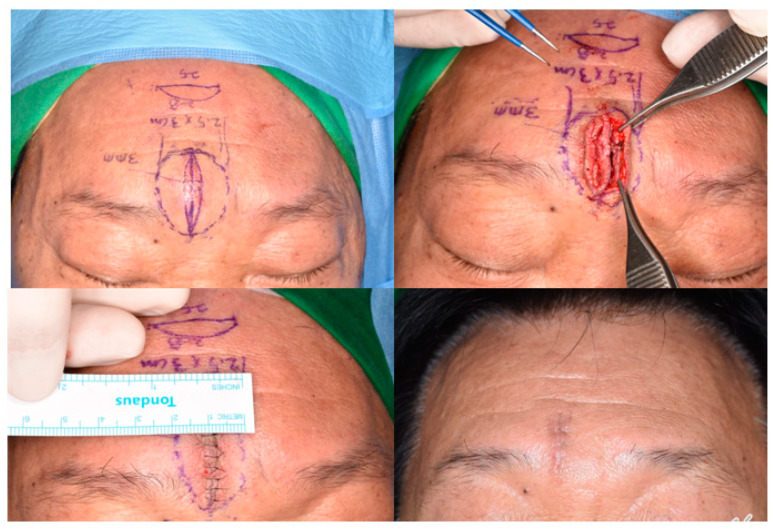
An elliptical skin-excision line is shown on the forehead lipoma. The skin expansion is approximately 3 mm compared to the base width. The excised skin and dermis are thinner than the adjacent skin. The result shows a fine linear scar without dog-ear deformity.

During the follow-up period, there were no reported cases of wound dehiscence, and no significant scar formation, such as hypertrophic scarring or depression, was observed at the surgical site, enhancing the esthetic appeal of the surgery. No patient experienced postoperative complications such as hematoma, seroma, or infection, as shown in Figure 4. The esthetic outcomes, as evaluated by the Cutometer and patient satisfaction survey, were favorable in all patients (Figure 5). Complications were minimal and included minor ecchymosis in 9 patients (1.5%) and transient skin numbness in 15 patients (2.2%), all of which resolved with conservative management.

Overall, mass excision with an elliptical skin attachment proved to be an effective, safe, and time-efficient technique for benign cutaneous and soft tissue masses, particularly for epidermal cysts. It provides satisfactory esthetic outcomes without significant postoperative complications.

### 3.3. mVSS

All scar areas were scored by a clinician using the mVSS. Pigmentation and pliability were the subcategories with the highest scores, with initial scores of 1.38 ± 0.82 and 1.85 ± 1.10 and a total score of 5.06 ± 3.97, which decreased at the 3-month follow-up by 0.88 ± 0.60 and 1.58 ± 1.12, respectively, with a total score of 3.67 ± 2.98 (Table 5).

## 4. Discussion

Various benign cutaneous and soft tissue tumors, including epidermal cysts, lipomas, dermoid cysts, and pilomatricomas, each have a different histological nature, and the removal of these conspicuous lumps without scar formation can be challenging [1]. Recently, more patients have been seeking not only complete mass removal but also minimal scar formation.

Conventional methods such as direct minimal excision and suction have numerous limitations, including the risk of incomplete removal and delayed healing, resulting in potentially unfavorable scarring [2,4,9,13,24,25,26]. In contrast, the current technique optimizes the skin healing capacity by excising the thin, less-vascularized skin areas and reducing complications such as infection, wound dehiscence, hematoma, and seroma formation.

Round or dome-shaped protruding masses make the overlying skin on the tumor thinner, especially at the central, most projected point. If the tumor expands, this condition can worsen. During surgery, the mass can rupture more easily, thereby causing remnant cells to spread through the wound and resulting in acute or late infection [9]. Additionally, wound margins require more retraction for clear sight, which can lead to margin sloughing [4,11]. 

If a mass grows larger, the central skin expands and becomes thin, making dissection without damaging the entire skin flap at each wound margin difficult. Moreover, the mass can easily rupture during dissection. Even if the thinned skin is well dissected without rupture, the thin, less-vascularized vulnerable skin flap from each wound margin has a weaker wound healing capacity than the normal, thick dermal areas [4,11,17]. However, if the thinned skin is removed together with the mass, the area that should be dissected is minimized, thereby reducing the risk of rupture. Before making the incision, the authors performed ultrasonography to reveal the shape, depth, and size of the mass and determine the thickness of the overlying skin. The incision line was marked over the thinned dermis based on the result, rendering a delicate incision, making it possible to prevent injury to the underlying mass. The attached skin was gradually removed in order for it to be grabbed without caution, which allowed for convenient handling during dissection. The operative time (mean, 17.48 ± 3.46 min) was significantly reduced, as compared to that with other methods reported by prior studies (*p* < 0.05), with operative times of 24.8 and 30.8 min for excisional and punch incision techniques performed for lesions measuring >2 cm, respectively [9]. This suggests the improved efficiency and potential cost-effectiveness of this procedure. 

Additionally, because the thin skin area was removed, the dermal thickness at the wound margin became thicker, which made burying the suture easier and ensured a more solid closure. Eventually, this led to rapid healing without any complications, including infection, wound dehiscence, hematoma and seroma formation, and recurrence, which can finally result in a favorable scar. 

The authors used both the Cutometer, a device that can measure the mechanical and viscoelastic characteristics of the skin based on a negative pressure-generation mechanism [18,19,20], and the mVSS to evaluate the clinical response to the treatment and its complications. Skin strength and elasticity can be determined by measuring the skin’s resistance to negative pressure and its ability to return to its original position [18,19,20]. A vacuum pump in the device creates negative pressure, drawing the skin into the probe’s aperture.

The mVSS was scored by the same physician in a similar manner to evaluate the overall difference between lesions. Among the available scar scores, the mVSS has suitable categories for capillary lesions [22]. Postoperative outcomes were overwhelmingly positive, with no notable cases of hypertrophic scarring or depression. This technique afforded a strong esthetic outcome, emphasizing its value for patients seeking to achieve minimal scarring. The minimal variation in Cutometer parameters between surgical and healthy skin demonstrates the preservation of skin elasticity, affirming the functional viability of the procedure, especially considering the relevance of esthetic outcomes in benign cutaneous masses in conspicuous locations [19,20,21,23].

The safety of this procedure was also highlighted by the lack of instances of postoperative hematoma, seroma, or wound infection and the resolution of all minor complications with conservative management. It is crucial to acknowledge that the focus on reducing postoperative complications considerably contributes to patient satisfaction and reduces the burden on healthcare resources [9,13,14,15,16].

The study presented evidence supporting the effectiveness and safety of elliptical skin excision with mass attachment for the removal of benign cutaneous masses. With a significantly shorter operative time than conventional methods and minimal postoperative complications, this method is a favorable alternative to traditional approaches, especially for the removal of epidermal cysts.

The success of this method has implications for setting new standards in the management of benign skin masses, emphasizing the relevance of a holistic approach that includes esthetic and psychological considerations, along with medical efficacy. However, the limitations of this study, such as the lack of a comparison with a control group undergoing conventional procedures, warrant further acknowledgment. Although the reduced operative time and minimal complications are promising, a more extensive comparative study would fortify the reliability of these results. For example, to analyze the correct result, objective diagnostic devices such as laser speckle contrast analysis (LASCA) can also be used [27,28,29].

Further research should focus on refining this technique and expanding its applicability to various types of masses and locations. Comparative studies using existing techniques would also enhance the validity of the presented outcomes, potentially exploring patient-centered outcomes such as pain, comfort, and satisfaction in more detail. Investigating the effectiveness, safety, and esthetic outcomes of this procedure in a diverse patient population would also be beneficial for generalizing the applicability of this technique.

## 5. Conclusions

In conclusion, elliptical skin excision using the mass attachment technique offers a promising alternative for benign cutaneous mass management, balancing efficiency, esthetic outcomes, and safety. This method appears to be particularly beneficial for lesions where esthetic considerations are crucial, with reduced scarring and preserved skin elasticity. Although these findings are promising, further comparative and diverse studies are imperative to consolidate the evidence for this technique. By extending our understanding of the surgical management of benign cutaneous masses, this research not only adds a novel technique to the existing repertoire but also accentuates the significance of addressing patients’ esthetic and psychological needs, along with the medical dimensions of care.

## Figures and Tables

**Table 1:** Parameters used to evaluate skin properties.

Parameters	Representation	Equivalent
R0	The final distension of the first curve	Uf
R1	The ability to return to the original state	Uf-Ua
R2	The overall elasticity of the skin, including creep and creep recovery	Ua/Uf
R3	The last maximum highest point of the last curve	Last Uf
R4	The last minimal lowest point of the last curve	Last R
R5	The net elasticity	Ur/Ue
R6	The ratio of viscoelastic to elastic extension, also called the viscoelastic ratio	Uv/Ue
R7	The ratio of elastic recovery to the total deformation	Ur/Uf
R8	The Ua of the first curve	First Ua
R9	The residual deformation at the end of the measuring cycle	R3–R0

**Table 2:** Patient demographics and mass characteristics.

	Male (*n* = 524)	Female (*n* = 456)	Total (*n* = 980)
Age (years)	58.1 (13–84)	43.2 (4–75)	53.5 (4–84)
Location, *n* (%)			
Head and neck	386 (73.7)	309 (67.8)	695 (70.9)
Trunk	90 (17.2)	83 (18.2)	173 (17.7)
Extremities	48 (9.2)	64 (14.0)	112 (11.4)
Pathology, *n* (%)			
Epidermal cyst	278 (53.1)	193 (42.3)	471 (48.1)
Lipoma	71 (13.5)	60 (13.2)	131 (13.4)
Pilomatricoma	22 (4.2)	34 (7.5)	56 (5.7)
Dermoid cyst	8 (1.5)	15 (3.3)	23 (2.3)
Other benign masses	145 (27.7)	154 (33.8)	299 (30.5)

**Table 3:** Mean operative time and mean mass size.

Surgical Time	
Size (mm) (long axis × short axis)	14.21 ± 7.50 × 11.33 ± 6.93
Incision line (mm) (length × width)	14.77 ± 7.15 × 2.17 ± 1.24
Surgical time (min)	17.48 ± 3.46

**Table 4:** Scar evaluation with the Cutometer^®^.

Cutometer MPA 580			
Parameters	Suture Line	Normal Skin	*p*-Value
R0	1.33 ± 0.30	1.52 ± 0.30	<0.05
R1	0.42 ± 0.25	0.39 ± 0.24	<0.05
R2	0.70 ± 0.15	0.75 ± 0.12	<0.05
R3	1.49 ± 0.30	1.65 ± 0.31	<0.05
R4	1.19 ± 0.32	1.24 ± 0.33	<0.05
R5	1.48 ± 0.31	1.27 ± 0.30	<0.05
R6	0.75 ± 0.17	0.65 ± 0.16	<0.05
R7	0.64 ± 0.21	0.52 ± 0.20	<0.05
R8	0.92 ± 0.26	1.13 ± 0.25	<0.05
R9	0.16 ± 0.02	0.12 ± 0.04	<0.05

**Table 5:** Scar evaluation with the mVSS at 1 and 3 months postoperatively.

mVSS			
Postoperative	1 Month	3 Months	*p*-Value
Vascularity	0.61 ± 0.75	0.35± 0.44	<0.05
Pigmentation	1.38 ± 0.82	0.88 ± 0.60	<0.05
Pliability	1.85 ± 1.10	1.58 ± 1.12	<0.05
Height	0.30 ± 0.47	0.41 ± 0.52	<0.05
Pain	0.76 ± 0.83	0.45 ± 0.30	<0.05
Total	5.06 ± 3.97	3.67 ± 2.98	<0.05

mVSS, modified Vancouver Scar Scale.

## Data Availability

The data presented in this study are available on request from the corresponding author.
